# Nutritional modulation of endogenous glucagon-like peptide-1 secretion: a review

**DOI:** 10.1186/s12986-016-0153-3

**Published:** 2016-12-09

**Authors:** Alexandra M. Bodnaruc, Denis Prud’homme, Rosanne Blanchet, Isabelle Giroux

**Affiliations:** 1 School of Human Kinetics, Faculty of Health Sciences, University of Ottawa, 35, University Private, Room 050F, K1N 6N5 Ottawa, ON Canada; 2 Institut de Recherche de l’Hôpital Montfort, Institut du savoir, 745 Montreal Road, Room 202, K1K 0T2 Ottawa, ON Canada; 3 School of Nutrition Sciences, Faculty of Health Sciences, University of Ottawa, 35 University Private, Room 050F, K1N 6N5 Ottawa, ON Canada; 4 School of Nutrition Sciences, Faculty of Health Sciences, University of Ottawa, 25 University Private, Room 116, K1N 6N5 Ottawa, ON Canada

**Keywords:** Diet, Appetite regulation, Blood glucose, Enteroendocrine L-cells, G-protein coupled receptors

## Abstract

**Background:**

The positive influences of glucagon-like peptide-1 (GLP-1) on blood glucose homeostasis, appetite sensations, and food intake provide a strong rationale for its therapeutic potential in the nutritional management of obesity and type 2 diabetes.

**Aim:**

To summarize GLP-1 physiology and the nutritional modulation of its secretion in the context of obesity and type 2 diabetes management.

**Findings:**

GLP-1 is mainly synthesized and secreted by enteroendocrine L-cells of the gastrointestinal tract. Its secretion is partly mediated by the direct nutrient sensing by G-protein coupled receptors which specifically bind to monosaccharides, peptides and amino-acids, monounsaturated and polyunsaturated fatty acids as well as to short chain fatty acids. Foods rich in these nutrients, such as high-fiber grain products, nuts, avocados and eggs also seem to influence GLP-1 secretion and may thus promote associated beneficial outcomes in healthy individuals as well as individuals with type 2 diabetes or with other metabolic disturbances.

**Conclusion:**

The stimulation of endogenous GLP-1 secretion by manipulating the composition of the diet may be a relevant strategy for obesity and type 2 diabetes management. A better understanding of the dose-dependent effects as well as the synergistic effects of nutrients and whole foods is needed in order to develop recommendations to appropriately modify the diet to enhance GLP-1 beneficial effects.

## Background

Type 2 diabetes (T2D) is a major public health concern due to its pandemic occurrence and its association with several physical and psychological comorbidities [1, 2], as well as with a decreased quality of life [3]. It is widely accepted that obesity, especially excessive fat accumulation in the abdominal area, is the most important predictor of T2D development. Indeed, it is estimated that 60 to 90% of T2D incidence is related to excessive weight gain and obesity [4]. After the onset of T2D, obesity also contributes to increasing the risks of comorbidities and overall mortality rates [5]. The global prevalence of overweight and obesity is expected to reach 57.8% by 2030 [6]. Following a similar trend, the worldwide prevalence of T2D has been projected to double between 2000 (2.2%) and 2030 (4.4%) [7].

Nutrition, along with physical activity and behavioural changes, are cornerstones of obesity and T2D management. Weight loss is induced by a state of sustained negative energy balance, which almost inevitably involves a reduction of energy intake [8]. From a purely thermodynamic perspective, a decrease in energy intake results in weight loss regardless of the type of dietary modifications through which it is achieved. Nonetheless, weight maintenance and improvements in cardiometabolic biomarkers are significantly impacted by the composition of the diet (i.e. macro- and micronutrient contents) [8]. One potential mechanism by which diet composition can influence these outcomes is through its influence on the secretion of gastrointestinal (GI) peptide hormones, including that of glucagon-like peptide-1 (GLP-1) [9]. The latter has been associated with a reduction of appetite and food intake, and appears to positively influence blood glucose homeostasis, by acting on the pancreas and the central nervous system [10].

The secretion of GLP-1 is partly mediated by the binding of nutrients to G-protein coupled receptors (GPCRs) expressed by enteroendocrine GI cells [11]. Therefore, manipulating the diet in a way to promote interactions with these receptors could increase GLP-1 secretion and enhance its beneficial effects [11]. This review focuses on GLP-1 physiology and the nutritional modulation of its secretion from enteroendocrine GI cells in the context of obesity and T2D management. It presents recent evidence on possible mechanisms by which specific foods, as well as nutrients and their by-products, could increase GLP-1 secretion, and subsequently influence appetite, food intake, and blood glucose control.

## Glucagon-like peptide-1 in the gut-brain-pancreas axis

### Synthesis, secretion, and metabolism

The GI tract accomplishes several functions, namely the digestion of food, the absorption of nutrients, and the secretion of digestive juices, mucus, and peptide hormones. The epithelium of the GI wall is composed of several cell types, including enteroendocrine cells which are a key component of the gut-brain-pancreas axis [12]. On their apical surface, enteroendocrine cells possess microvilli expressing several GPCRs that are binding to nutrients and other substrates present in the GI lumen [12]. Enteroendocrine cells can be divided into several subcategories depending on their distribution throughout the GI tract, their GPCRs’ expression and their secretory profile. GLP-1 is synthesized and secreted by enteroendocrine L-cells which are expressed over a large portion of the GI tract, starting in the proximal small intestine and progressively increasing in density down to the distal part of the colon. GLP-1 is stored in secretory granules of L-cells until its secretion is triggered, and then uses endocrine and neuronal routes to exert its functions in the pancreas and central nervous system [10]. In addition to L-cells, GLP-1 is synthesized to a lesser extent by neurons of the nucleus tractus solitarius (NTS) of the brainstem [13, 14].

GLP-1 is produced in two major active forms, namely GLP-1 (7-36 amide) and GLP-1 (7-37 amide), and is resulting from the differential processing of its precursor proglucagon [10]. Its synthesis appears to be attributed to tissue-specific expression of pro-hormone convertase 1 and 3 which cleave proglucagon [15]. Proglucagon is a 160-amino acid inactive precursor of several peptide hormones, including glucagon, oxyntomodulin and GLP-1 [16]. Proglucagon encoding gene is expressed in the intestine, the pancreas, as well as the central nervous system [16]. Several studies have confirmed that this gene produces identical messenger ribonucleic acid (mRNA) transcripts in these major expression sites, but is translated and processed differently, thus producing different bioactive peptides depending on the expressing tissue [17–19].

In humans, blood concentrations of GLP-1 generally range between 5 pmol/L and 15 pmol/L in fasting state and increase two- to four-folds after food ingestion [10]. More specifically, GLP-1 blood concentrations rise within 15 minutes after food ingestion and reach a pick after approximately 60 minutes [10]. In the second hour, GLP-1 concentrations start to decrease gradually until the next prandial episode [10]. Postprandial GLP-1 secretion is influenced by both neuroendocrine and nutritional factors, and exhibits a two-phase release profile that is in fact very similar to that of insulin. The initial phase of its secretion, which is detectable 10 to 15 minutes after food ingestion, is thought to be mostly influenced by neuroendocrine factors, and, to a lesser extent, by the interaction of nutrients with L-cells in the proximal small intestine. Conversely, the second phase of GLP-1 secretion, which occurs 30 to 60 minutes postprandially, is mostly influenced by the arrival of nutrients in the distal part of the small intestine and the colon [20]. Nutrients and their by-products bind to GPCRs and activate intracellular pathways that ultimately trigger GLP-1 exocytosis from L-cells’ secretory granules [10]. When secreted, GLP-1 can activate vagal afferent neural fibres, as well as diffuse into nearby capillaries and then reach the systemic circulation through the portal vein [10]. In the bloodstream, GLP-1 is highly susceptible to the catalytic activity of the enzyme dipeptidyl-peptidase IV (DPP-IV). The latter cleaves two NH_2_-terminal amino acids of the active forms of GLP-1 (i.e. 7-36 amide, 7-37 amide) leading to the production of their biologically inactive forms (i.e. 9-36 amide, 9-37 amide) [21–23]. As a result, GLP-1’s half-life is very short – about 1 to 2 min [22, 23]. Animal and *in vitro* studies have shown that less than 25% of the newly secreted bioactive GLP-1 reaches the liver intact [22, 23]. Further catalytic activities take place in the liver, and consequently, only approximately 10-15% of the newly secreted GLP-1 reaches the systemic circulation in its active forms [22, 23].

### Action in the pancreas

One of the best-known and probably most important effects of GLP-1 is its ability to stimulate insulin secretion in response to carbohydrate consumption [24–27]. In pancreatic β-cells, GLP-1 activates intracellular pathways that increase intracellular calcium concentrations and subsequently leads to insulin exocytosis from secretory granules [24, 25]. GLP-1 has also been shown to promote insulin gene transcription and biosynthesis [28]. GLP-1 seems to be responsible for nearly half of the total postprandial insulin secretion [29]. This process, known under the name of *“incretin effect”*, is defined as the differential augmentation in insulin secretion observed after oral glucose intake, compared to intravenous glucose administration resulting in the same blood glucose concentrations [30].

GLP-1 is partly fulfilling its actions in pancreatic β-cells through an endocrine pathway by directly interacting with its receptors (GLP-1Rs). Indeed, competitive inhibition of GLP-1Rs of pancreatic β-cells in rodents and humans resulted in a significant reduction of insulin secretion, as well as in impaired glucose tolerance [26, 27]. Nonetheless, the glucose-lowering effects of GLP-1 are not solely related to its insulinotropic effect, but also to its strong inhibition of glucagon secretion [10, 31]. This effect seems to be partly mediated by the increased insulin and somatostatin secretion from pancreatic islets occurring during the postprandial period, as well as by GLP-1 direct interaction with its receptors on pancreatic α-cells [32–34]. In rodents, prolonged administration of GLP-1 also appears to promote pancreatic β-cell growth by increasing their proliferation and decreasing apoptosis [35–38].

### Action in the central nervous system

In addition to its action in the pancreas, GLP-1 plays a role in both homeostatic and non-homeostatic regulations of food intake, which occur in distinct areas of the central nervous system [39, 40]. The homeostatic regulation of food intake, related to short- and long-term energy status, is mainly taking place in the hypothalamus and NTS, areas which convey and integrate numerous peripheral signals [39, 40]. The hypothalamus contains several interconnected nuclei, including the arcuate nucleus (ARC), the paraventricular nucleus (PVN), as well as the dorso-medial nucleus (DMN) [39, 40]. Due to its anatomical position, the ARC plays a critical role in transmitting peripheral information related to energy and nutrient status to other central structures. Indeed, the ARC is situated in the medio-basal area of the hypothalamus, where the blood-brain barrier is highly permeable, and thus likely has a greater exposition to circulating factors [39]. The ARC contains two distinct populations of neurons: 1) the orexigenic neuropeptide Y (NPY) and agouti-related peptide (AgRP) expressing neurons, and 2) the anorexigenic pro-opiomelanocortine (POMC), as well as cocaine and amphetamine-regulated transcript (CART) expressing neurons [39, 40]. Additionally, POMC is a precursor of the α-melanocyte stimulating hormone (α-MSH), a neuropeptide with potent anorexigenic effects [39, 40]. Upon release, POMC and α-MSH activate neurons of the PVN, which inhibits food intake [39, 40]. Conversely, by inhibiting PVN neurons’ activation, NPY and AgRP stimulate food intake [39, 40]. Opposite to the PVN, DMN neurons are activated by NPY and AgRP, promoting food intake, and are inhibited by POMC and α-MSH, reducing food intake [39, 40].

GLP-1Rs are widely expressed in the hypothalamus, with the highest expression being described to be in POMC/CART neurons of the ARC, followed by DMN neurons [13, 14, 41–44]. A small quantity of GLP-1 appears to diffuse through the blood-brain barrier and directly bind to its receptors on POMC/CART and NPY/AgRP neurons [45]. In rodents, the peripheral administration of a GLP-1 analogue has been shown to directly activate neurotransmission in POMC/CART neurons, while exerting an inhibitory action in NPY/AgRP neurons [46, 47]. Nonetheless, in reason of its short half-life, endogenous GLP-1 released from L-cells is likely mostly acting on the central nervous system by indirectly stimulating neurons of the NTS and ARC via the activation of vagal afferent neurons [48]. Indeed, GLP-1Rs have been identified on neurons of the nodose ganglion of the vagus nerve [43, 49], and the importance of this pathway in the regulation of food intake has been confirmed in rats, where vagal deafferentation decreased the effects of intraperitoneally administered GLP-1 [50, 51]. While the exact mechanisms and their relative contributions to the observed effects remain to be fully elucidated, the GLP-1’s implication in the homeostatic regulation of food intake is well established. In rodents, acute intraperitoneal, subcutaneous or intravenous administration of GLP-1 and GLP-1 analogues have constantly been shown to reduced meal size, as well as cumulative food and energy intakes [51–56]. Similarly, in humans, acute intravenous administration of GLP-1 and GLP-1 analogues decreased appetite, hunger and food intake, and increased fullness and satiety sensations [57–62].

In addition to physiological energy needs, food intake is driven by non-homeostatic central pathways involved in reward processing and reward-motivated behaviours [63, 64]. The palatability of food is a crucial determinant of the decision to eat. As a result, highly palatable food, typically high in energy, lipids, and simple carbohydrates, can trigger food intake in the absence of physiological energy needs [63, 64]. Several central structures, such as the orbitofrontal cortex, insula, amygdala, and striatum play an important role in the processing and evaluation of food cues [65]. Increased activation of these areas of the central nervous system in response to visual and olfactory food cues (referred to as *“anticipatory food reward”*) is associated with increased craving for highly palatable foods [66, 67]. Furthermore, upon food ingestion, dopaminergic neurons send projections from the ventral tegmental area to the nucleus accumbens and other forebrain areas [63, 64]. Dopamine release in these areas of the brain is well correlated with meal pleasantness in healthy individuals with a normal body mass index [68]. Activation of these areas in response to food consumption is referred to as *“consummatory food reward”*. Reduced consummatory food reward is associated with compensatory overeating [65–67, 69, 70]. GLP-1Rs have been identified in areas of the brain involved in anticipatory and consummatory food reward processing, and neurons of the NTS also share dense neuronal connections with these areas [71, 72]. Recent pre-clinical and clinical studies suggest a role of GLP-1 in the modulation of food reward processing. More specifically, the exogenous administration of GLP-1 and GLP-1 analogues appears to influence dopamine neurotransmission in several central areas, and has been associated with decreased anticipatory food reward, increased consummatory food reward and decreased intake of hyperpalatable foods [65, 69, 70, 73–77].

## Glucagon-like peptide-1 as a target for obesity and type 2 diabetes management

The positive influences of GLP-1 on many metabolic disturbances associated with T2D, as well as key determinants of weight loss and weight maintenance, make it a therapeutic target with good potential. GLP-1 has been studied in relation to obesity and T2D pathophysiology and treatment. While few studies have shown contradictory results, it is generally well accepted that obesity and metabolic changes occurring with the development of T2D are associated with a decline in the postprandial secretion of GLP-1 from L-cells [78–83]. Overtime, this change in GLP-1’s secretion can contribute to further weight gain and adversely impact T2D progression. As a potential strategy to enhance GLP-1actions, several researchers have investigated the metabolic effects of intravenous administration of GLP-1 analogues. Interestingly, when receiving the same dose of a GLP-1 analogue, individuals with obesity and T2D exhibited metabolic and appetite responses that were very similar to their healthy counterparts [57–59, 62, 84, 85]. This finding suggests that sensibility to GLP-1’s action is maintained, which is contrary to several other peptide hormones involved in energy and blood glucose homeostasis [86, 87]. Consequently, targeting GLP-1Rs’ activation with GLP-1 analogues and increasing GLP-1 half-life in the bloodstream became an area of interest for the management of obesity and T2D. This led to the development of two classes of pharmacologic agents, namely GLP-1Rs agonists and DDP-IV inhibitors [88, 89]. GLP-1Rs agonists are widely used for blood glucose management in individuals with T2D and have recently been approved to use for weight management in the United States [88–90]. Similarly, DDP-IV inhibitors are used alone or in combination with other pharmaceutical agents to inhibit the catabolic deactivation of endogenous GLP-1 [88, 89]. Compared to other commonly used antihyperglycemic drugs such as sulfonylureas and thiazolidinedione, pharmaceutical agents targeting GLP-1’s action are associated with lower risks of hypoglycaemia, and have the advantage of promoting weight loss as well as potentially preventing, or at least delaying the progressive decrease in pancreatic β-cell function which generally requires increasing drug dosage [35–38, 89, 91]. Most frequent side effects reported with the use of these agents include GI discomfort, nausea and vomiting [88, 89].

Another relevant, yet less studied, strategy to increase GLP-1’s action is to promote its endogenous secretion from L-cells. This could potentially be achieved through pharmacological and dietary approaches. The main critic of dietary approaches is that dietary changes may not lead to a sufficient increase in GLP-1 blood concentration to promote beneficial physiological actions such as improvements in insulin secretion and blood glucose concentrations. Indeed, GLP-1Rs agonist mimic supraphysiological blood concentrations of GLP-1 and have a far longer half-life, varying between 1.5 hours and 5 days depending on the agent [89]. Nonetheless, GLP-1Rs agonists only exert their effects through an endocrine route, while endogenous GLP-1 employs both endocrine and neuronal routes, and therefore such high concentrations may not be needed. In fact, changes in GLP-1 blood concentrations after bariatric surgery suggest that a rise within physiologically normal blood concentrations may be sufficient to promote beneficial metabolic effects. Several studies have shown that Roux-en-Y gastric bypass increases GLP-1 blood concentrations up to fourfold 6 months to 1 year after the procedure [92, 93]. This increase in GLP-1 concentrations is associated with improvements in dietary choices and intake, further excess weight loss, improved glycaemic control, and often T2D resolution [92, 94]. Le Roux et al. [92] showed that individuals who underwent Roux-en-Y gastric bypass within 36 months had significantly higher GLP-1 blood concentrations 30 minutes following a meal ingestion (47.4 ± 11.4 pmol/L) compared to subjects with morbid obesity (13.5 ± 6.9 pmol/L). Postprandial GLP-1 concentrations observed in subjects who underwent Roux-en-Y gastric bypass were within physiologically normal GLP-1 concentration ranges, and therefore it is plausible that promoting a similar increase in GLP-1 postprandial secretion with dietary approaches would promote similar beneficial actions on food intake, glycaemic control and weight management over time.

## Nutritional modulation of glucagon-like peptide-1 secretion

The nutrient composition of ingested food and meals varies considerably between individuals and within the same individual over the course of the day. In this regard, macronutrient composition of the diet has been shown to influence appetite and metabolic responses, including glycaemia and insulinemia [95, 96]. One possible mechanism to explain the differential effects of macronutrients on these parameters is the modulation of GLP-1 secretion by their catabolic by-products. As described above, L-cells’ GPCRs bind several products of food and macronutrients’ breakdown, including monosaccharides [97–102], short-chain fatty acids (SCFAs) [103–109], medium and long-chain fatty acids [110, 111] , as well as amino acids and peptides [112–116], leading to GLP-1 secretion.

### Nutrients and single-nutrient foods

#### Monosaccharides

Upon ingestion, digestible carbohydrates undergo enzymatic degradation and are absorbed in the form of glucose, and to a lesser extent in the form of galactose and fructose. Glucose absorption by enterocytes as well as glucose-mediated GLP-1 secretion from L-cells appear to be mediated by the sodium glucose transporter-1 (SGLT-1), a membrane transport protein expressed in the small intestine [97–102]. Moriya and colleagues [98] investigated the mechanisms underlying glucose-stimulated GLP-1 secretion by administering glucose and Phloritzine, a competitive inhibitor of SGLT-1, in the small intestine of mice. While glucose administration alone acutely increased circulating GLP-1, co-administration of glucose and Phloritzine blocked first-phase glucose-induced GLP-1 secretion [98]. Similarly, compared to controls, SGLT-1 knockout mice had decreased first-phase GLP-1 secretion and developed glucose and galactose malabsorption [99]. This is explained by the fact that glucose binding to SGLT-1 induces the closure of ATP-sensitive potassium channels, leading to membrane depolarization and to a rise in intracellular calcium concentrations [117]. Collectively, this work outlines the importance of SGLT-1 in luminal monosaccharide absorption, as well as glucose-mediated GLP-1 secretion in the proximal small intestine. The characterization of GLP-1’s secretion as being bi-phasic led researchers to investigate the prolonged effect the pharmacological inhibition of SGLT-1 in rodents and humans [100–102]. While also confirming an inhibition of first-phase GLP-1 secretion and an increase in luminal glucose concentrations, Powell and al. [100] found an increase in second-phase GLP-1 secretion and a decrease in blood glucose excursions over a 6-hour postprandial period in mice. These results were confirmed in two clinical studies conducted in healthy adults, as well as in adults with T2D [101, 102]. The increase in second-phase GLP-1 secretion observed in these studies suggests that SGLT-1 inhibition enhances other mechanisms promoting GLP-1 secretion from the distal part of GI tract. As proposed by the authors, possible explanations include an enhanced opportunity for interactions with L-cells as carbohydrates move down the GI tract during digestion, as well as an increased fermentation of undigested carbohydrates in the colon which could raise the production of SCFAs [100–102]. Concordant with the latter hypothesis, Powell and al. [100] observed a decrease in the pH of the caecum – which could indeed be an indicator of increased bacterial fermentation – in SGLT-1 knockout mice as well as mice that received a SGLT-1 inhibitor.

#### Non-digestible carbohydrates and short-chain fatty acids

In the colon, non-digestible carbohydrates undergo fermentation, leading, depending on their type, to the production of various amounts of SCFAs [118–121]. SCFAs are carboxylic acids that contain fewer than 6 carbons, the most abundant ones being acetate, butyrate and propionate [122]. In humans, SCFAs concentrations range from ~130 mmol/L in the caecum to ~80 mmol/L in the descending colon [122, 123]. Acetate appears to be the most abundant SCFA in the colon, followed by propionate and butyrate, with an overall colonic molar ratio of 50-60:15-20:10-20, respectively [122, 123].

Fermentable dietary fiber and their metabolites, the SCFAs, seem to promote GLP-1 secretion from L-cells by interacting with the free fatty acid receptors 2 and 3 (FFAR2, FFAR3) [103–109]. An *in vitro* study showed that propionate was the most potent agonist of both FFAR2 and FFAR3, that acetate was more active and selective on FFAR2, and that butyrate was more active on FFAR3 than FFAR2 [105]. In colonic cell cultures, physiological concentrations of acetate, butyrate and propionate have been shown to stimulate GLP-1 secretion [124]. The role played by FFAR2 and FFAR3 in the SCFA-induced GLP-1 secretion was confirmed by demonstrating the loss of postprandial GLP-1 secretion in FFAR2 and FFAR3 knockout intestinal cells [124]. Similar results were found *in vivo*, where propionate-induced GLP-1 secretion was lost in FFAR2 knockout rodents [125]. Additionally, Chambers and colleagues [126] recently showed that acute targeted delivery of propionate in the colon through an inulin-propionate ester supplement acutely stimulated GLP-1 secretion following a standard breakfast meal and reduced energy intake at a buffet-style lunch in adults with overweight or obesity. Concordant with the acute effects of GLP-1 on appetite sensations and food intake, daily delivery of propionate in the colon over 6 months also reduced body weight, abdominal fat and hepatic lipid accumulation as assessed with magnetic resonance imagery and spectroscopy [126].

The effects of diets rich in fermentable fiber on GLP-1 secretion have also been investigated in animal models and humans [127, 128]. Indeed, the main way to increase colonic SCFA concentrations in humans is through non-digestible carbohydrate consumption. The results from six experimental studies assessing the impact of fiber on GLP-1 secretion in animal models and humans are summarized in Table 1. Compared to a standard control diet, the consumption of a diet enriched in fermentable fiber for 50 days increased GLP-1 concentrations in the proximal colon, which was associated with an increased expression of proglucagon in colonic L-cells [129]. Fermentable fiber also decreased food intake, weight gain, as well as blood triglyceride concentrations and triglyceride accumulation in the liver [129]. Similarly, while also increasing GLP-1 blood concentrations and proglucagon expression in the colon, consumption of a diet rich in resistant starch for 10 days increased peptide tyrosine-tyrosine (PYY) blood concentrations and colonic expression in rats [130]. This result is not surprising as the anorectic PYY is also synthesized and released from L-cells, and its secretion could be stimulated by similar mechanisms as that of GLP-1. In healthy humans, a crossover pilot study showed that the daily supplementation with 16 g of fermentable fiber for a two-week period increased postprandial satiety, and decreased hunger, prospective food consumption as well as energy intake over 24 hours following the intake of a standard breakfast meal [131]. As GLP-1 blood concentrations were not assessed in this study, it is not known whether these effects were mediated by an increase in its secretion or through other mechanisms [131]. The effects of fermentable fiber in rodent models of diabetes were comparable to those found in healthy animals. Indeed, consumption of a diet enriched with fermentable fiber for 4 to 6 weeks increased GLP-1 concentrations in the proximal colon and portal vein, as well proglucagon expression in the proximal colon [132, 133]. In streptozotocin-treated diabetic rats, the fiber-enriched diet also up-regulated pro-hormone convertase 1 expression in the proximal colon and increased pancreatic β-cell mass which potentially mediated the improvement in glucose tolerance and the observed increase in insulin and C-peptide blood concentrations [132]. In mice with high-fat diet-induced diabetes, these effects were diminished with the use of a GLP-1R antagonist and were completely lost in a GLP-1R knockout group of rats, therefore confirming the importance of GLP-1 interaction with its receptors for exerting its beneficial effects [133].

**Table 1:** Chronic^a^ effects of non-digestible carbohydrates on glucagon-like peptide-1 (GLP-1) secretion and associated outcomes

Studies	Experimental model	Dietary intervention	Main outcomes measured
Cani 2005a [129]	〉 Male Wistar rats	Oligofructose (10% of diet^b^)	↑ GLP-1 concentrations in the proximal and medial colon ↑ Proglucagon mRNA concentrations in the caecum and proximal colon ↓ Triglyceride blood and hepatic concentrations ↓ Food and energy intake ↓ Body weight gain
Cani 2005b [132]	〉 Streptozotocin-treated diabetic rats	Oligofructose (10% of diet^b^)	↑ Proglucagon mRNA concentrations in the proximal colon ↑ Pro-hormone convertase 1 mRNA concentrations in the proximal colon ↑ GLP-1 concentrations in the portal vein ↑ Glucose tolerance ↑ Insulin and C-peptide blood concentrations ↑ Pancreatic β-cell mass ↓ Food and energy intake
Cani 2005c [131]	〉 Healthy men and women	Oligofructose (16 g/day)	↓ Energy intake over 24 hours ↑ Satiety ↓ Hunger ↓ Prospective food consumption
Cani 2006 [133]	〉 Male C57BL/6 J mice with high-fat feeding-induced diabetes	Oligofructose (10% of diet^b^)	↑ GLP-1 concentrations in the proximal colon ↑ GLP-1 concentrations in the portal vein ↑ Proglucagon mRNA concentrations in the proximal colon ↑ Blood and pancreatic insulin concentrations ↓ Fasting and postprandial blood glucose concentrations
Cani 2007 [134]	〉 Male Wistar rats	Oligofructose (10% of diet^b^)	↑ GLP-1 concentrations in the portal vein and proximal colon ↑ Proglucagon mRNA concentrations in the proximal colon ↑ L-cell number in the proximal colon ↑ Neurogenin-3 and Neuro-D mRNA concentrations in the proximal colon ↓ Food and energy intake ↓ Body weight gain
Zhou 2008 [130]	〉 Sprague-Dawley rats 〉 Streptozotocin-treated C57BL/6 J mice	Resistant starch (53% of diet^b^)	↑ GLP-1 blood concentrations ↑ PYY blood concentrations ↓ Variation in blood glucose and insulin concentrations ↑ Proglucagon and PYY colonic mRNA concentrations ↑ Glucose tolerance in diabetic mice

*GLP-1* Glucagon-like peptide-1, *mRNA* Messenger ribonucleic acid, *PYY* Peptide tyrosine-tyrosine

^a^ 10 days - 1 year interventions

^b^ weight : weight proportion

Overall, results from these studies suggest some pathways linking fermentable fiber to its positive effects on food intake, body weight gain and blood glucose homeostasis via an increased secretion of GLP-1. Fermentable fiber and SCFAs produced by their colonic fermentation appear to increase GLP-1 synthesis by up-regulating the expression of proglucagon [129, 132–134], as well as that of pro-hormone convertase 1 [132], which, as discussed above, is responsible for its cleavage [15]. Cani and colleagues [134] also found an increased proximal colon’s L-cell number with fermentable fiber consumption, which could be another possible explanation for the increased GLP-1 synthesis and secretion. The increase in L-cell number was also associated with an enhanced expression of transcription factors that are critical for the differentiation of intestinal stem cells into enteroendocrine cells, namely neuregenin-3 and neuro-D [134].

#### Free fatty acids

The majority of dietary lipids are triglycerides, which are composed of a glycerol molecule and three fatty acids [135]. Upon ingestion, triglycerides undergo emulsification by bile salts in the duodenum, hydrolysis by lipases, and are absorbed by enterocytes in the form of glycerol and free fatty acids [135].

Free fatty acids, such as unsaturated long-chain fatty acids, are potent stimulators of GLP-1 release through interactions with free fatty acid receptors 1 and 4 (FFAR1, FFAR4) [110, 111]. Substrate binding to FFAR1 and FFAR4 activate phospholipase C, leading to inositol triphosphate mediated calcium release from the endoplasmic reticulum into the cytosol [136]. The secretion of GLP-1 has been shown to be increased by unsaturated long-chain fatty acids. The main outcomes of nine experimental studies assessing the impact of lipid consumption on GLP-1 secretion and blood concentrations are summarized in Table 2. Thomsen and colleagues (1999, 2003) were the first ones to assess GLP-1 responses following a meal containing olive oil in healthy adults or adults with T2D [137, 138]. Compared with a meal containing butter, which is high in saturated fatty acids (SFAs), the ingestion of the olive oil containing meal resulted in higher postprandial GLP-1 blood concentrations [137, 138]. However, no significant acute difference in blood glucose, nor insulin blood concentrations was observed [137, 138]. In a subsequent study conducted in rodents, the prolonged consumption of an olive oil-enriched diet resulted in an increased GLP-1 secretion, which coincided with a higher glucose-stimulated insulin secretion, as well as an improved glucose tolerance at the 36^th^ day of the intervention [139]. Comparable results were found in Streptozotocin-treated diabetic rats, where the intake of a diet rich in monounsaturated fatty acids (MUFAs) from olive oil for 50 days increased GLP-1 blood concentrations, decreased weight gain and improved insulin sensitivity [140]. In humans with abdominal obesity and insulin resistance, Paniagua and colleagues [141] showed that ingestion of a Mediterranean diet rich in olive oil for 28 days resulted in significantly higher postprandial GLP-1 blood concentrations. Compared to a diet high in SFAs, consumption of the Mediterranean diet also improved insulin sensitivity, an effect which could have mediated the observed decrease in insulin secretion as well as fasting and postprandial blood glucose concentrations [141].

**Table 2:** Acute^a^ and chronic^b^ effects of monounsaturated fatty acids (MUFAs) and omega-3 polyunsaturated fatty acids (PUFAs) on glucagon-like peptide-1 (GLP-1) secretion and associated outcomes

Studies	Experimental model	Dietary intervention	Main outcomes measured
Thomsen 1999 [137]	〉 Healthy men and women	MUFAs from olive oil^a^ (80 g)	↑ GLP-1 blood concentrations ↑ Glucose-insulinotropic peptide blood concentrations
Thomsen 2003 [138]	〉 Men and women with T2D	MUFAs from olive oil^a^ (80 g)	↑ GLP-1 blood concentrations ↓ Triglyceride blood concentrations ↑ High-density lipoprotein cholesterol blood concentrations
Hirasawa 2005 [136]	〉 Male C57/B6 rats	α-linolenic acid (0.1 μmol)^a^	↑ GLP-1 blood concentrations ↑ Insulin blood concentrations
Prieto 2005 [139]	〉 Wistar rats	MUFAs from olive oil^b,c^	↑ GLP-1 blood concentrations ↑ Glucose-stimulated insulin secretion ↑ Glucose tolerance
Cancelas 2006 [140]	〉 Streptozotocin-treated diabetic rats	MUFAs from olive oil^b,c^	↑ GLP-1 postprandial secretion ↑ Insulin sensitivity ↓ Weight gain
Adachi 2006 [142]	〉 Male C57BL/6 J mice 〉 Diabetic male NSY mice 〉 C3H/He mice	α-linolenic acid (0.3 μmol)^a^	↑ GLP-1 blood concentrations ↑ Insulin blood concentrations ↓ Blood glucose concentrations
Paniagua 2007 [141]	〉 Insulin resistant men and women	MUFAs from an olive-oil containing Mediterranean diet (23% of total energy intake)^b^	↑ Postprandial GLP-1 concentrations ↑ Insulin sensitivity ↓ Postprandial insulin secretion ↓ Fasting and postprandial blood glucose concentrations
Tanaka 2008 [143]	〉 Male Wistar rats	α-linolenic acid (3 μmol)^a,b^	↑ GLP-1 blood concentrations ↑ pancreatic β-cell number and mass
Cheshmehkani 2015 [144]	〉 Male Sprague-Dawley rats	Fish or flaxseed oil^b,d^ (10% of diet)	↑ FFAR4 expression in the colon ↓ TNFα blood concentrations

FFAR4: Free fatty acid receptor 4; GLP-1: Glucagon-like peptide-1; MUFAs: Monounsaturated fatty acids; SCFAs: Short-chain fatty acids; TNFα: Tumour necrosis factor α

^a^ Acute effect (5 minutes post-consumption)

^b^ Chronic effect (28 to 50 days of consumption)

^c^ Specific MUFA content not known

^d^ weight : weight proportion

In addition to the beneficial effects of MUFAs, some studies showed that the colonic administration of the polyunsaturated α-linolenic acid acutely increased GLP-1 and insulin blood concentrations, and decreased blood glucose concentrations in healthy and diabetic rat models [142]. Similarly, while also resulting in increased GLP-1 blood concentrations, long-term administration of α-linolenic acid has been shown to increase β-cell proliferation in rodents [143]. As GLP-1 has been shown to decrease apoptosis, as well as to increase neogenesis of pancreatic β-cells, the authors hypothesized that the increased β-cell proliferation was mediated by the increase in GLP-1 concentrations induced by α-linolenic acid ingestion [143]. A recent study showed that fish oil and flax seed oil, which are a source of α-linolenic acid, increased FFAR4 expression in rodents’ colon and decreased the expression of the pro-inflammatory tumour necrosis factor α (TNFα) [144].

Taken together, these studies suggest that, with similar energy contents, diets that are richer in MUFAs or omega-3 polyunsaturated fatty acids (PUFAs) than in SFAs, could increase GLP-1 secretion from L-cells, which may be a mediator for the increase in insulin secretion, insulin sensitivity, β-cell proliferation, as well as the improved glucose tolerance observed in animal models and in humans.

#### Peptides and amino acids

Dietary proteins are generally described as the most satiating nutrient, an effect which may be partly mediated by the stimulation of anorexigenic GI peptides secretion, including that of GLP-1 [145–147]. Upon ingestion, proteins are broken down by acid hydrolysis and proteases to produce peptones, tripeptides, dipeptides and single amino acids. Specifics mechanisms responsible for protein-induced GLP-1 secretion are relatively poorly understood, and the optimal profile of proteins’ breakdown products remains to be elucidated. Nonetheless, two pathways by which products of protein breakdown seem to stimulate GLP-1 secretion is through their binding to the calcium-sensing receptors (CaSR) and the class C, group 6, subtype A GPCR (GPCR-C6A) which are expressed on L-cells. CaSR binds to a variety of amino acids including phenylalanine, tryptophan, asparagine, arginine, glutamine and histidine, as well as to peptones, tri- and dipeptides [112–116]. *In vitro*, their binding to CaSR stimulated GLP-1 secretion, and this effect was abolished with the use of a CaSR inhibitor [112–116]. Oya and colleagues [148] have shown that GPCR-C6A binds to the basic amino acids arginine, lysine and ornithine. The stimulatory effect of these amino acids on GLP-1 secretion was abolished in GPCR-C6A knockout intestinal cells [148].

### Mixed-nutrient foods

In contrast with single nutrients and single-nutrient foods (e.g. oil), more complex foods containing a mix of nutrients are more representative of what humans consume and could allow targeting a combination of enteroendocrine pathways that would synergistically enhance GLP-1 secretion. In regards to T2D management, clinical guidelines recommend carbohydrate intake from high-fiber foods such as vegetables, fruit, legumes and whole grains, while limiting SFAs intake and promoting that of MUFAs and omega-3 PUFAs [149, 150], all of which have been associated to different extents with an increased GLP-1 secretion.

Some experimental studies conducted with healthy individuals have examined the effects of several specific foods on glycemic response, subjective appetite sensations as well as energy intake at a subsequent meal [151–168]. The main findings of these studies are summarized in Table 3. Several researchers have investigated the effects of high-fiber grain products, which could enhance GLP-1 secretion by binding to SGLT-1, FFAR2 and FFAR3. In this regard, two recent studies compared appetite responses after isoenergetic breakfast meals containing ready-to-eat breakfast cereals (low-fiber) or oatmeal (high-fiber) among healthy young adults [151, 152]. Compared to ready-to-eat breakfast cereals, oatmeal (66.8 g) increased fullness, and reduced hunger, desire to eat, as well as subjective prospective food intake [151, 152]. Furthermore, oatmeal decreased energy intake at the following meal [152]. Likewise, in adults with insulin resistance, daily consumption of a high wheat-fiber breakfast cereal (test group, 24 g/day of fiber from the cereal) for one year, significantly increased colonic SCFAs production, as well as GLP-1 blood concentrations compared to low-fiber breakfast cereals (control group, 0.5 g/day of fiber from the cereal) [165]. More specifically, at 9 months into the intervention, acetate and butyrate plasma concentrations were already significantly higher in the test group [165]. Along the same lines, Nilsson and colleagues [166] showed that consumption of high-fiber barley kernel-based bread for 3 days was associated with increased fasting GLP-1 blood concentrations, as well as increased postprandial PYY blood concentrations in healthy adults. These changes in GI peptides concentrations were associated with improved insulin sensitivity and decreased postprandial blood glucose concentrations in healthy individuals [166]. The fact that the authors found an increase in breath hydrogen and SCFAs blood concentrations, suggests that the positive effects of the barley kernel-based bread were mediated by an increased SCFAs production triggered by the colonic of dietary fiber [166].

**Table 3:** Summary of experimental studies assessing the effects of mixed-nutrient foods on glucagon-like peptide-1 (GLP-1) secretion and associated outcomes

Studies	Subjects		Dietary intervention		Main outcomes measured
	N	Characteristics	Food	Duration	
Rebello 2013 [151]	48	〉 Healthy^a^	Oatmeal^b^ (66.8 g)	4 h	↑ Fullness ↓ Hunger ↓ Desire to eat ↓ Prospective food consumption
Rebello 2016 [152]	48	〉 Healthy	Oatmeal^b^ (66.8 g)	4 h	↑ Fullness ↓ Hunger ↓ Desire to eat ↓ Prospective food consumption ↓ Energy intake at the next meal
Freeland 2010 [165]	28	〉 Hyperinsulinemia	High-fiber cereal (24 g/day)	1 year	↑ Acetate and butyrate blood concentrations ↑ GLP-1 blood concentrations
Nilsson 2015 [166]	20	〉 Healthy	Barley kernel-based bread^c^ (100 g of available starch per day)	3 days	↑ PYY postprandial blood concentrations ↑ GLP-1 fasting blood concentrations ↑ Fasting and postprandial breath hydrogen concentrations ↑ Total SCFAs and acetate fasting blood concentrations ↓ Blood glucose postprandial concentrations ↓ Insulin postprandial blood concentrations
Jenkins 2006 [156]	15	〉 Healthy	Raw almonds^b^ (60.0 g)	4 h	↓ Blood glucose postprandial concentrations ↓ Insulin postprandial blood concentrations ↓ Oxydative damage to proteins measured in blood serum
Josse 2007 [167]	9	〉 Healthy	Raw almonds^c^ (30, 60 and 90 g)	4 h	↓ Blood glucose postprandial concentrations^d^
Jenkins 2008 [157]	27	〉 Hyperlipidemia	Raw almonds- (37.0 and 73.0 g)	1 month	↓ 24-hour insulin secretion
Mori 2011 [159]	14	〉 Impaired glucose tolerance	Raw almonds^c^ (42.5 g)	8 h	↓ Blood glucose concentrations ↑ Insulin blood concentrations ↓ Non esterified fatty acid blood concentrations after a second meal ↑ Fullness
Kendall 2011 [153]	10	〉 Healthy	Pistachios^b^ (28.0, 56.0 and 84.0 g)	2 h	↓ Blood glucose concentrations^d^
Kendall 2014 [154]	20	〉 Metabolic syndrome	Pistachios^c^ (85.0 g)	3 h	↑ GLP-1 postprandial blood concentrations ↑ Glucose-insulinotropic peptide postprandial blood concentrations ↓ Blood glucose postprandial concentrations ↑ Insulin postprandial blood concentrations
Reis 2013 [168]	30	〉 Obese women	(a) Whole peanuts^c^ (42.5 g) (b) Peanut butter^c^ (42.5 g)	8 h	↓ Blood glucose concentrations after second standard isocaloric meal (b) ↑ Insulin postprandial blood concentrations (a,b) ↓ Non esterified fatty acid blood concentrations after second meal (b) ↑ PYY postprandial blood concentrations (b) ↓ Desire to eat following the second meal (a,b)
Parham 2014 [155]	48	〉 Type 2 diabetes	Pistachio^d^ (50.0 g per day)	12 weeks	↓ Fasting blood glucose concentrations ↓ Percentage of glycated blood haemoglobin ↓ Systolic blood pressure ↓ Body mass index ↓ C-reactive protein blood concentrations
Ratliff 2010 [160]	21	〉 Healthy men	Whole egg^b^ (3)	24 h	↓ Blood glucose postprandial concentrations ↓ Ghrelin postprandial blood concentrations ↓ Insulin postprandial blood concentrations ↓ Hunger ↑ Satisfaction ↓ Energy intake at lunch and over 24 hours
Pombo-Rodrigues 2011 [162]	31	〉 Healthy	Whole eggs^b^ (2)	4 h	↑ Fullness ↓ Desire to eat ↓ Prospective food consumption
Vander Wal 2005 [161]	30	〉 Overweight women	Whole eggs^b^ (2)	36 h	↑ Satiety ↓ Energy intake at the next meal and over 24 and 36 hours
Liu 2015 [163]	28	〉 Healthy children and adolescents	Eggs^b^	3 h	↑ PYY postprandial blood concentrations
Wien 2013 [164]	26	〉 Overweight	Fresh avocado^b^ (50.0 to 90.0 g)	3 h	↓ Insulin postprandial blood concentrations ↓ Desire to eat ↑ Satisfaction

*GLP-1* Glucagon-like peptide-1, *PYY* Peptide tyrosine-tyrosine, *SCFAs* Short-chain fatty acids

^a^ Unless otherwise mentioned, study samples include adults of both sexes

^b^ Isocaloric test and control meals, however, not marched for macronutrient contents

^c^ Test and control meals matched for carbohydrate content only

^d^ In a dose-dependent matter

The addition of foods with a high protein, MUFAs and fiber content, such as almonds (30.0 to 90.0 g) or pistachios (28.0 to 85.0 g) to a high-carbohydrate meal has also been shown to improve postprandial glycemic responses in a dose-dependent manner [153–159, 167]. Jenkins and colleagues [156] also found a decrease in insulin secretion in healthy adults and in adults with hyperlipidemia following acute and prolonged almond consumption. Similarly, daily intake of 50.0 g of pistachios for 12 weeks decreased C-reactive protein blood concentrations, systolic blood pressure, and body mass index in adults with T2D [155]. In women with obesity, consumption of peanuts (42.5 g) or peanut butter (42.5 g) as part of a standard breakfast meal increased postprandial insulin blood concentrations and decreased desire to eat following a standard lunch meal [166]. Additionally, peanut butter significantly increased postprandial PYY blood concentrations and decreased postprandial blood glucose concentrations following the standard lunch meal [166]. Since GLP-1 and PYY are co-released from L-cells [12], it is plausible that peanut butter may also promote postprandial GLP-1 secretion. Only three of these studies assessed GLP-1 blood concentrations [154, 159, 168]. Mori et al. [159] found no significant differences in GLP-1 blood concentrations following the addition of 42.5 g of almonds to a carbohydrate-containing meal, which could be due to the small sample size of the study. Indeed, with a larger sample size, Kendall et al. [154] found higher GLP-1 and glucose insulinotropic peptide blood concentrations following the addition of 85.0 g of pistachios to a carbohydrate-containing meal in adults with metabolic syndrome. As Reis et al. [168] showed no statistically significant difference in GLP-1 blood concentrations with the addition of 42.5 g of peanuts or peanut butter to a carbohydrate-containing meal, it is also possible that higher quantities of nuts are necessary to induce a sufficient rise in GI hormone secretion.

Consumption of eggs (2 to 3), which are high in protein and also contain MUFAs, for breakfast or lunch, has been shown to improve subjective postprandial appetite sensations [160–163]. Furthermore, when compared to a bagel breakfast, consumption of a breakfast containing eggs (3) in adult men was associated with a lower postprandial blood glucose concentrations, decreased hunger, and reduced energy intake in the next 24 hours [160]. Men also reported higher subjective satisfaction after eating eggs [160]. While GLP-1 blood concentrations were not measured in these studies, Li and al. [163] found a significant increase in postprandial blood concentrations of PYY in adolescents following ingestion of an egg-containing breakfast compared to an isocaloric bagel breakfast. One study also showed that adding avocado (50 to 90 g), which is high in fiber and MUFAs, to a high-carbohydrate isocaloric meal improved subjective satiety sensation and satisfaction [164].

## Discussion and Conclusion

In light of this literature review, it is evident that manipulating the composition of the diet in order to promote GLP-1 secretion represents a promising lifestyle strategy for obesity and T2D management. The therapeutic potential of GLP-1 has already been established as several pharmaceutical agents promoting its effects are successfully used for blood glucose management in individuals with T2D, and for body weight management in individuals with obesity [87, 89]. In comparison with the use of GLP-1Rs agonists and DPP-IV inhibitors, targeting endogenous pathways through dietary modifications has the added benefits of potentially stimulating the release of other anorexigenic gut peptides (e.g. PYY), promoting beneficial changes in several markers of cardiometabolic health such as helping normalize blood lipid profile and blood pressure, as well as avoiding side effects associated with medication. Furthermore, as DPP-IV pharmacological action depends on the bioavailability of endogenous GLP-1 into the blood-stream, combining the use of this drug type with a diet promoting GLP-1 secretion could promote DPP-IV inhibitors’ action and potentially allow the use of lower doses. Therefore, it would be worthwhile to investigate the relationship between diet composition and DPP-IV inhibitors’ efficacy in future studies.

When summarizing existing studies on the modulation of endogenous GLP-1 secretion from enteroendocrine L-cells by single nutrients and their by-products (e.g. non digestible carbohydrates and SCFAs), single-nutrient foods (e.g. olive oil, fish oil) as well as mixed-nutrient foods, it appears that the nutritional modulation of GLP-1 secretion involves several GPCRs expressed on L-cells. Furthermore, specific mechanisms for the increase in GLP-1 synthesis and secretion in L-cells include the up-regulation of proglucagon expression [129, 132–134], as well as that of post-transcriptional factors (e.g. pro-hormone convertase 1) [132]. The synthesis and secretion of GLP-1 may also be enhanced by promoting the differentiation of intestinal stem cells into L-cells [134].

In comparison with single nutrients, mixed-nutrient foods theoretically constitute a more promising and practical treatment option as they can allow targeting several enteroendocrine pathways. The main limitation of existing studies assessing the effects of specific foods is that it is impossible to distinguish the specific impact of macronutrient types (e.g. MUFAs versus SFAs) from the impact of the amount of the intake *per se* because none of the experimental meals were matched for macronutrient content. Furthermore, several studies [154, 156, 161, 167, 168] did not use isocaloric test and control meals. In these studies, the test meal was contained more calories which leads to question whether the observed effects is to be attributed to the tested foods or the energy content of the meal. These limitations call for more rigorous experimental designs to confirm the effects of these foods on GLP-1’s and other gut hormone’s secretion. Future studies should also aim to gain a better understanding of dose-specific effects of single nutrients, mixed nutrients, specific foods and food combinations in humans. There is also a need to compare the effects of food ingestion on GLP-1 secretion in healthy adults versus adults with metabolic disturbances such as metabolic syndrome, impaired glucose tolerance, and T2D. Well-designed randomised control trials should be planned to evaluate the impact of foods promoting GLP-1 secretion on the progression from impaired glucose tolerance to frank T2D. Lastly, efforts should be made to identify ranges of postprandial GLP-1 concentrations that are associated with beneficial metabolic effects acutely and in the long-term. Such knowledge advances could help individuals modify their dietary habits in a way that obesity and T2D could be better managed and maybe prevented.

## Abbreviations

AgRP: Agouti-related peptide
ARC: Arcuate nucleus
CART: Cocaine and amphetamine-regulated transcript
CaSR: Calcium-sensing receptor
DMN: Dorso-medial nucleus
DPP-IV: Dipeptidyl-peptidase IV
FFAR1-4: Free fatty acid receptor 1-4
GI: Gastrointestinal
GLP-1: Glucagon-like peptide-1
GLP-1R(s): Glucagon-like peptide-1 receptor(s)
GPCR(s): G-protein coupled receptor(s)
GPCR-C6A: Class C group 6, subtype A G-protein coupled receptor
mRNA: Messenger ribonucleic acid
MUFA(s): Monounsaturated fatty acid(s)
NPY: Neuropeptide Y
NTS: Nucleus tractus solitarius
POMC: Pro-opiomelanocortine
PUFA(s): Polyunsaturated fatty acid(s)
PVN: Paraventricular nucleus
PYY: Peptide tyrosine-tyrosine
SFA(s): Saturated fatty acid(s)
SGLT-1: Sodium glucose transporter-1
T2D: Type 2 diabetes
TNFα: Tumour necrosis factor α
α-MSH: α-melanocyte stimulating hormone
